# Pulmonary Tuberculosis, Etiological and Therapeutic

**Published:** 1891-06

**Authors:** 


*Pulmonary Tuberculosis, Etiological and Therapeutic.* By R. W. PHILIP, M.A., M.D., F.R.S.E. Edinburgh: YOUNG J. PENTLAND. 1891.

The author of this essay discusses the *modus operandi* of the tubercle bacillus in leading towards death, and endeavours to prove that the result is due to its power of elaborating new products which are afterwards absorbed. He summarises a large number of observations on frogs, mice, and rabbits, in which the toxic effects of an extract of phthisical sputum and their antagonism by atropine were demonstrated. He proceeds to make certain therapeutic observations, and suggests a general division of phthisis into three stages:

1. Catarrhal, previous to inoculation with the bacillus.
2. Invasion by the tubercle bacillus.
3. Elaboration and absorption, when toxic effects are induced, with characteristic clinical features.

We think that the existence of a pretubercular stage such as is meant by No. 1 is by no means proved; and we should be sorry to encourage the far too common idea that tubercular phthisis is simply bronchitis for many months, and then becomes tuberculous by inoculation into a suitable soil. Doubtless tuberculous mischief may exist in the lung for long periods anterior to the manifestation of catarrhal symptoms, and at the very earliest stage of many cases, even anterior to the manifestation of physical signs of disease in the lung, the sputum may be found to contain multitudes of bacilli. On the other hand, the chronic catarrhal patients, with bronchitis recurring for many years, emaciated and apparently an easy prey to bacillary disease, may live an indefinite time, and escape tubercular inoculation altogether. Catarrhal processes are a very importantpart of the phenomena of phthisis, but we are inclined to look upon the invasion of the bacillus as the first stage and the catarrh as a secondary product.

The author emphasises the utility of intra-tracheal injection, and he prefers eucalyptus oil in olive oil, or the following:

| | | |
|---|---|---|
| Chloroform. pur. ... ... ... ... | | ♏xxx. |
| Balsam. Peru.... ... ... ... ... | | ʒij. |
| Olei Eucalypti ... ... ... ... | | ʒij. |
| Ol. Ricini... ... ... ... ... .. | ad. | ℥j. |

Sig. Thirty mimins for dose once or twice daily.

The object of treatment is *not so much the actual death of the bacilli present*, but rather the saturation of the medium with such agents as may be supposed, or may be proved experimentally, to be *prejudicial to the vital activity of the organisms ;* for, as has been said, "the life of the bacillus is difficult, easily discouraged by unfavourable circumstances, like an aphis by an easterly wind."

As regards the treatment of the stage of absorption, the author's experimental observations give good ground for a modern endorsement of Trousseau's belief that atropine exerts a specific action on the lung-tissue in phthisis.

